# The clinical implications and molecular features of intrahepatic cholangiocarcinoma with perineural invasion

**DOI:** 10.1007/s12072-022-10445-1

**Published:** 2022-11-22

**Authors:** Xian-Long Meng, Jia-Cheng Lu, Hai-Ying Zeng, Zhen Chen, Xiao-Jun Guo, Chao Gao, Yan-Zi Pei, Shu-Yang Hu, Mu Ye, Qi-Man Sun, Guo-Huang Yang, Jia-Bin Cai, Pei-Xin Huang, Lei Yv, Lv Zhang, Ying-Hong Shi, Ai-Wu Ke, Jian Zhou, Jia Fan, Yi Chen, Xiao-Yong Huang, Guo-Ming Shi

**Affiliations:** 1 Department of Liver Surgery and Transplantation, Zhongshan Hospital, Fudan University, Shanghai, 200032 China; 2 Liver Cancer Institute, Fudan University, Shanghai, 200032 China; 3 Key Laboratory of Carcinogenesis and Cancer Invasion, Ministry of Education of the People’s Republic of China, Shanghai, 200032 China; 4 Department of Pathology, Zhongshan Hospital, Fudan University, Shanghai, 200032 China; 5 Clinical Research Unit, Institute of Clinical Science, Zhongshan Hospital of Fudan University, Shanghai, 200032 China

**Keywords:** Perineural invasion, Intrahepatic cholangiocarcinoma, Sympathetic nerve, KRAS, Pathology feature, Metastasis prone niche, Adjuvant therapy, Overall survival, Relapse-free survival, Bioinformatics

## Abstract

**Background:**

Perineural invasion (PNI) is associated with metastasis in malignancies, including intrahepatic cholangiocarcinoma (ICC), and is correlated with poor prognosis.

**Methods:**

The study included three large cohorts: ZS-ICC and TMA cohorts from our team, MSK cohort from a public database, and a small cohort named cohort 4. Prognostic implications of PNI were investigated in MSK cohort and TMA cohort. PNI-related genomic and transcriptomic profiles were analyzed in MSK and ZS-ICC cohorts. GO, KEGG, and ssGSEA analyses were performed. Immunohistochemistry was used to investigate the relationship between PNI and markers of neurons, hydrolases, and immune cells. The efficacy of adjuvant therapy in ICC patients with PNI was also assessed.

**Results:**

A total of 30.6% and 20.7% ICC patients had PNI in MSK and TMA cohorts respectively. Patients with PNI presented with malignant phenotypes such as high CA19-9, the large bile duct type, lymph node invasion, and shortened overall survival (OS) and relapse-free survival (RFS). Nerves involved in PNI positively express tyrosine hydroxylase (TH), a marker of sympathetic nerves. Patients with PNI showed high mutation frequency of KRAS and an immune suppressive metastasis prone niche of decreased NK cell, increased neutrophil, and elevated PD-L1, CD80, and CD86 expression. Patients with PNI had an extended OS after adjuvant therapy with TEGIO, GEMOX, or capecitabine.

**Conclusion:**

Our study deciphered the genomic features and the immune suppressive metastasis-prone niche in ICC with PNI. Patients with PNI showed a poor prognosis after surgery but a good response to adjuvant chemotherapy.

**Graphical abstract:**

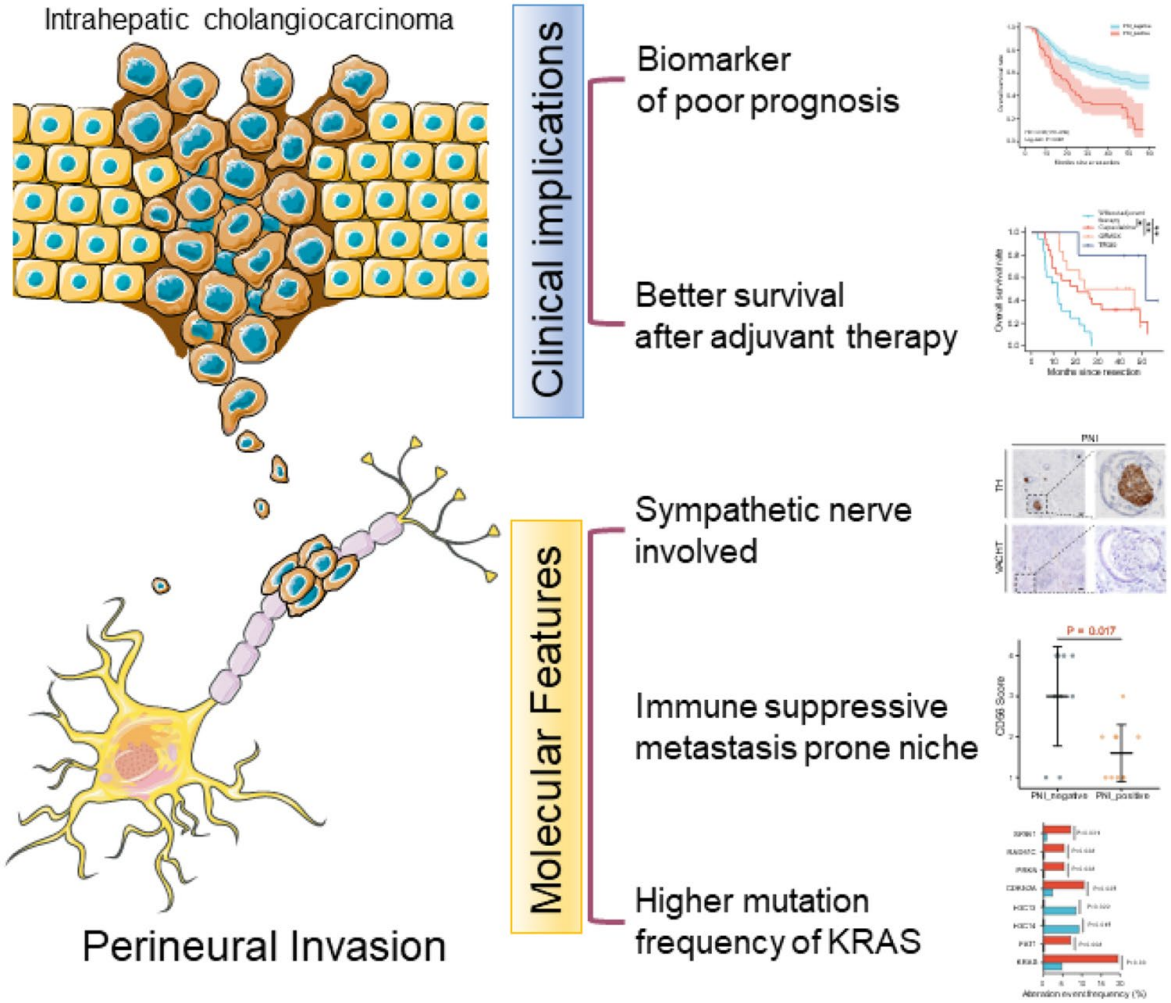

**Supplementary Information:**

The online version contains supplementary material available at 10.1007/s12072-022-10445-1.

## Background

Intrahepatic cholangiocarcinoma (ICC) is the second most common primary liver cancer, originating from the secondary and higher intrahepatic bile duct branches [1]. The prognosis of patients with ICC remains poor owing to early metastasis and lack of effective treatment strategies [2].

Perineural invasion (PNI) in cancer was first reported in the nineteenth century [3]. PNI is defined as the appearance of tumor cells along the nerves and/or within the epineural, perineural, and endoneuria regions of the neuronal sheath, with cancer cells surrounding at least one-third of the nerves [3]. Recently, the clinical significance of PNI in malignancies has been noticed. PNI was observed in over 80% of pancreatic ductal adenocarcinoma (PDAC) cases and was found to be an early event of tumorigenesis in preclinical and clinical models [4, 5], reprogramming the immune microenvironment with decreased CD8^+^ T and Th1 cells, and elevated Th2 cells [6]. In addition, PNI has been considered as an independent prognostic factor in several cancers, including gastric cancer [7], cervical cancer [8], gallbladder cancer [9], breast cancer [10], prostate cancer [11], hepatocellular carcinoma (HCC) [12], and ICC [13]. Considering the significant implications of the prognosis of cancers, the molecular characteristics of PNI and its potential targets are of great significance. In particular, recent evidence has established a link between the nervous system and the immune microenvironment, wherein the nerve fibers were observed to colocalize with subclones of lymphocytes in PDAC, including CD20^+^ B cells, CD4^+^, CD8^+^ T cells, and CD21^+^ follicular dendritic cells [14]. The colocalization of nerve fibers with immune cells provides direct evidence for neuroimmunomodulation in malignancies [15]. Preliminary evidence has shown that PNI can reprogram the immune microenvironment through cholinergic signaling in PDAC [6]. Unfortunately, the molecular profile of PNI and the relationship between PNI and the immune microenvironment in ICC remain unclear.

In this study, we investigated the role of PNI in two independent cohorts of patients with ICC and confirmed the contribution of PNI as an unfavorable prognostic factor post-surgery in these patients. We found that the PNI in case of ICC is mainly derived from the sympathetic nerve (SNS). We also demonstrated that patients with PNI presented with an immune suppressive metastasis prone niche, high frequency of KRAS mutation, and better survival after adjuvant therapy.

## Materials and methods

### Patients and clinical samples

Study participants included four cohorts. Cohort 1: 255 ICC patients with RNA-seq and genomic data from our group (named as ZS-ICC cohort) [16]. Cohort 2: 186 ICC patients with genomic data, PNI and clinical information from MSK cohort [17]. Cohort 3: 309 patients with ICC who underwent curative resection between 2013 and 2017 at Zhongshan Hospital, Fudan University (named as TMA cohort). Enrolled patients met the following criteria [18]: (1) pathologically confirmed ICC; (2) ≥ 3 months of relapse-free survival (RFS) after resection; (3) had not undergone anti-tumor treatment before surgery; and (4) had complete medical records and follow-up data available. Patients were stratified by a tumor-node-metastases (TNM) stage system according to the American Joint Committee on Cancer (AJCC) 8th edition. The histological grade of ICC was based on World Health Organization Criteria. Tumor samples and adjacent liver tissue samples were collected, formalin-fixed, and paraffin-embedded. The last follow-up was on December 31, 2020. Cohort 4: 19 pathologically confirmed patients with ICC who underwent curative resection from March 2016 to May 2016 at Zhongshan Hospital, Fudan University.

### Tissue microarrays, immunohistochemistry and hematoxylin–eosin (H&E) staining

The tissue microarrays (TMAs) were constructed as previously described and immunohistochemistry (IHC) was performed as our previous study [19]. Anti-human rabbit monoclonal antibodies for monoamine oxidase A (MAO-A) (1:200; #ab126751, Abcam, Cambridge, UK), anti-human mouse monoclonal antibodies for beta-tubulin III (1:100; #4466S, CST, Massachusetts, USA), anti-human rabbit monoclonal antibodies for tyrosine hydroxylase (TH) (1:300; #58844S, CST, Massachusetts, USA), anti-human mouse monoclonal antibodies for vesicular acetylcholine transporter (VACHT) (1:100; #MA5-27,662, ThermoFisher, Waltham, USA) and anti-human rabbit monoclonal antibodies for CD56 (1:200; #99746S, CST, Massachusetts, USA) were used as primary antibodies to detect the expression of MAO-A, TH, VACHT and CD56, respectively. Automated digital pathological slice scanner, KF-PRO-120 (KONFOONG biotech international CO.LTD., Ningbo, China) and NanoZoomer S360 (Hamamatsu Photonics CO.LTD., Beijing, China), were used to scan images of IHC slides, and slides were photographed by Digital slices view software K-Viewer (KONFOONG biotech international CO.LTD., Ningbo, China) and NDP-viewer (Hamamatsu Photonics CO.LTD., Beijing, China).

### Evaluation of MAO-A and CD56 expression

TMAs of cohort 3 consisting of two paired spots of tumor tissue and peri-tumor tissue from one patient were used to investigate the expression of MAO-A. For each spot, five represent visions were randomly chosen and the optical density (OD) and area for each vision were calculated by Image-Pro Plus (version 6.0, Media Cybernetics, Inc., China). MAO-A staining score was counted as OD/area and defined the average score of total ten visions as the expression of MAO-A of each patient. Cut off values were calculated through X-tile [20]. Cohort 4 was used to investigate the expression of CD56 as followed. Five representative spots were chosen for each patient and the number of CD56 positive cells were counted. The average number of positive cells of five spots was considered as the CD56 expression level of each patient. The score was defined as numbers of CD56 positive cells: (1) 0 or 1: 1 point; (2) 2 or 3: 2 points; (3) 4 to 10: 3 points; (4) more than 10: 4 points.

### Statistical and bioinformatic analysis

Statistical analyses were performed with SPSS 25.0 (Chicago, IL, USA), and GraphPad Prism 8 software (La Jolla, CA, USA). Values are presented as median (range) or mean ± standard deviation (SD). Unpaired Student’s *t*-test, Fisher’s exact test, Chi-square test and the Wilcoxon rank-sum test were used to compare differences between groups. The Kaplan–Meier method was used to construct the survival and recurrence curves. Cox proportional hazards model analysis was used to analyzing the correlation between variables and ICC patient prognosis. Statistical tests were two-tailed, and *p*-value < 0.05 was considered significant. Differentially expressed gene analysis was operated in R (version 4.1.2, R foundation for statistical, Vienna, Austria). Codes used are available on request. Gene Ontology (GO), Kyoto Encyclopedia of Genes and Genomes (KEGG) were operated on https://www.xiantao.love/products/apply/. Single sample Gene Set Enrichment Analysis (ssGSEA) was performed on ImmuCellAI [21].

## Results

### PNI is an unfavorable prognostic factor for patients with ICC post-operation

**Fig. 1:**
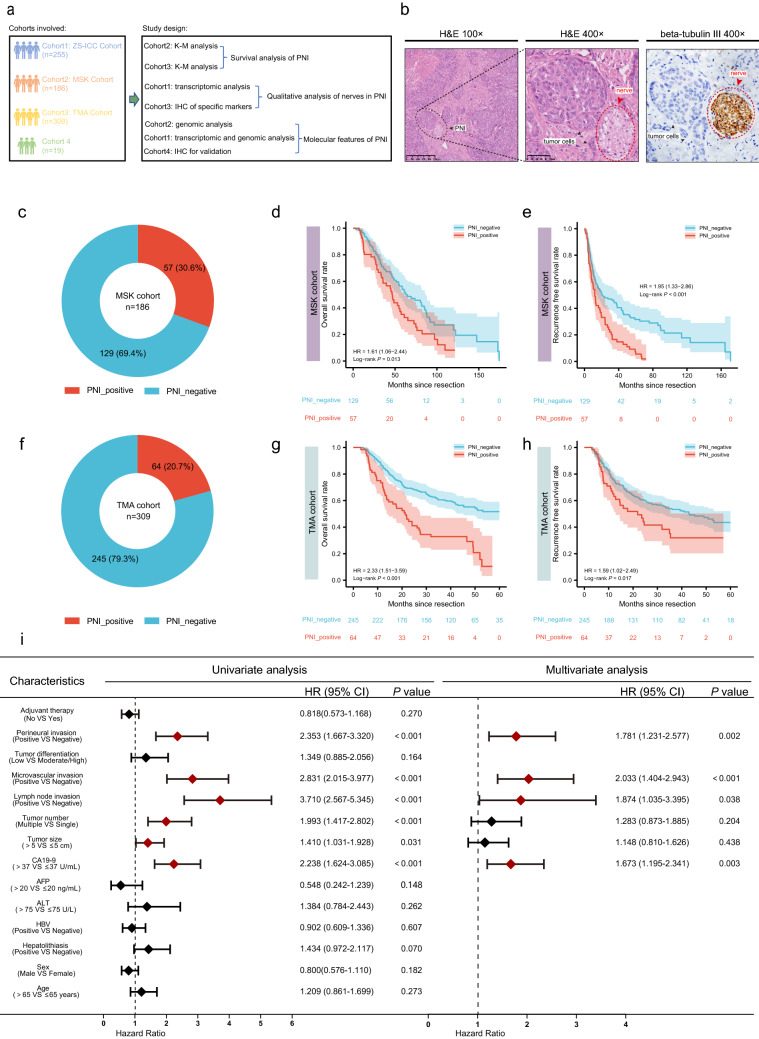
PNI is an unfavorable prognostic factor for patients with ICC post-operation. (**a**) Cohorts involved and study design of this article: ZS-ICC cohort (n = 255), cohort 4 and TMA cohort (n=309) are from Zhongshan hospital, Shanghai, China; MSK cohort is from a public database. (**b**) Representative images of H&E staining and beta-tubulin III staining of PNI in TMA cohort.(**c**) Fan chart of the components of MSK cohort: PNI positive cases accounted for 30.6% (n=57) of MSK cohort. (**d**) K–M analysis of OS between patients with and without PNI in MSK cohort (HR = 1.61, 1.06–2.44, P = 0.013). (**e**) K–M analysis of RFS between patients with and without PNI in MSK cohort (HR = 1.95, 1.33–2.86, P < 0.001). (**f**) Fan chart of the components of the TMA cohort: PNI positivity accounted for 20.7% (n = 64) of TMA cohort. (**g**) K–M analysis of OS between patients with and without PNI in TMA cohort (HR = 2.33, 1.51–3.59, P < 0.001).(**h**) K–M analysis of RFS between patients with and without PNI in TMA cohort (HR = 1.59, 1.02–2.49, P = 0.017). (**i**) Forest illustration of univariate and multivariate analyses of OS in TMA cohort (HBV: hepatitis B virus; ALT: alanine aminotransferase; AFP: alpha fetoprotein; CA19-9: carbohydrate antigen199; HR: hazard ratio; CI:confidence interval)

The MSK cohort (n = 186) and the TMA cohort (*n* = 309) were used to investigate the role of PNI in ICC (Fig. 1a, Table S3). PNI was evaluated by H&E staining and further confirmed by IHC of beta-tubulin III in TMA cohort (Fig. 1b) and was observed in 30.6% and 20.7% of patients in the MSK and TMA cohorts, respectively (Fig. 1c. f). The overall survival (OS) of patients with PNI was evidently shorter than that of patients without PNI (HR = 1.61, *p* = 0.013; HR = 2.33, *p* < 0.001, respectively) (Fig. 1d, g). Similarly, the relapse-free survival (RFS) of patients with PNI was conspicuously lower than that of those without PNI (HR = 1.95, log-rank *p* < 0.001; HR = 1.59, log-rank *p* = 0.017, respectively) (Fig. 1e, h). We also analyzed the relationship between PNI and other clinicopathological features and found that PNI positivity was significantly correlated with high CA19-9 level, the large duct type ICC and lymph node invasion in two independent cohorts. (Tables 1, S1). Recently, a multicenter study reported that PNI is a powerful and independent predictor of recurrence and survival in ICC [22]. Consistently, univariate and multivariate analyses of TMA cohort revealed that PNI (HR = 1.781, *p* = 0.002), microvascular invasion (HR = 2.033, *p* < 0.001), lymph node invasion (HR = 1.874, *p* = 0.038), and CA19-9 level (HR = 1.673, *p* = 0.003) are independent risk factors for OS in ICC patients (Fig. 1i). According to the 5th WHO classification, the large duct type ICC exhibited poorer prognosis than those with the small type, and PNI is usually observed in the large duct type ICC. To rule out the influence of duct type, multivariate analyses was performed and confirmed PNI was a negative risk factor (HR = 2.391, *p* < 0.001) independent of the duct type (Fig. S1a). Similarly, K-M analyses confirmed patients with PNI showed poorer prognosis either in the large duct type group (HR = 4.25, *P* < 0.001) or the small duct type group (HR = 1.72, *p* = 0.015) (Fig. S1b, c).

**Table 1:** Baseline demographics and clinicopathological variables among patients of ICC with and without PNI in TMA cohort

	PNI		*p* value
	Negative	Positive	
Age (year)			
≤ 65	173	48	0.489
> 65	72	16	
Sex			
Female	98	20	0.200
Male	147	44	
Hepatolithiasis			
Negative	203	51	0.555
Positive	42	13	
HBV infection			
Negative	44	14	0.475
Positive	201	50	
ALT (U/L)			
≤ 75	230	57	0.181^#^
> 75	15	7	
AFP (ng/mL)			
≤ 20	229	61	0.773^#^
> 20	16	3	
CA19-9 (U/mL)			
≤ 37	139	25	0.012*
> 37	106	39	
Tumor size (cm)			
≤ 5	127	44	0.015*
> 5	118	20	
Tumor number			
Single	188	48	0.771
Multiple	57	16	
Duct type			
Small	172	36	0.034*
Large	73	28	
Lymph node invasion			
Negative	214	49	0.031*
Positive	31	15	
Microvascular invasion			
Negative	196	46	0.160
Positive	49	18	
TNM stage			
I/II	193	38	0.001*
III	52	26	
Tumor differentiation			
Low	33	10	0.675
Moderate/High	212	54	
Adjuvant therapy			
No	59	18	0.506
Yes	186	46	

*HBV* hepatitis B virus, *ALT* alanine aminotransferase, *AFP* alpha fetoprotein, CA19-9: carbohydrate antigen199;

^#^Fisher’s exact test; *p < 0.05

### PNI derived from sympathetic nerves in ICC

**Fig. 2:**
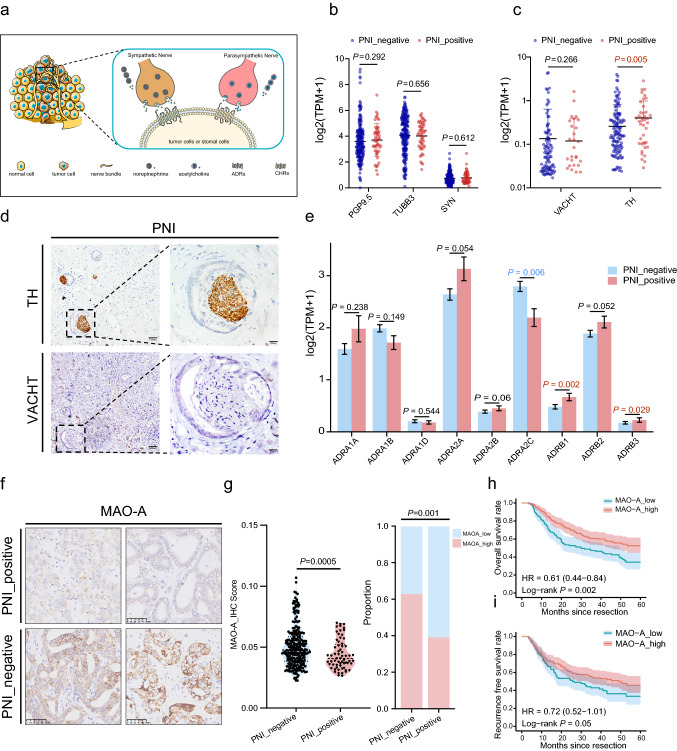
PNI derived from sympathetic nerves in ICC. (**a**) Graphical exhibition of the interactions between automatic nerves and tumor. (**b**) Dot plot of PGP9.5 (P = 0.292), TUBB3 (P = 0.656), and SYN (P = 0.612) of ZS-ICC cohort; Unpaired t-test was used. (**c**) Dot plot of mRNA expression of VACHT (P = 0.266) and TH ( = 0.005) in ZS-ICC cohort; Unpaired t-test was used. (**d**) Representative IHC image of TH positive nerves involved in patients with PNI in TMA cohort. (**e**) Bar plot of ADRs mRNA expression between patients with and without PNI in ZS-ICC cohort; Unpaired t-test was used. (**f**) Representative images of IHC staining of MAO-A in TMA cohort. (**g**) Comparison of MAO-A expression between patients with and without PNI in TMA cohort: picture on the left is the violin plot of the MAO-A IHC score between PNI positive and PNI negative patients, and unpaired t-test was used to compare the differences (P = 0.0005); picture on the right is the bar plot of the proportion of different levels of MAO-A in PNI positive cases and PNI negative cases, and chi-square test was used (P = 0.001). (**h**) K–M analysis of OS between patients with high and low MAO-A expression in TMA cohort (HR = 0.61, 0.44–0.84, P = 0.002). (**i**) K–M analysis of RFS between MAO-A high and low expression patients in the TMA cohort (HR = 0.72, 0.52–1.01, P = 0.05)

Sympathetic nerve system (SNS) or parasympathetic nerve system (PNS)-derived nerve fibers have been reported to participate in the progress of tumors [23] (Fig. 2a), thereby propelling the search for the origin of PNI in ICC. We investigated the expression of representative pan-neural, SNS, and PNS markers in ZS-ICC cohort at the mRNA level. Among the five markers, significant upregulation of TH (*p* = 0.005), a biomarker of SNS [24], was observed in patients with PNI from the ZS-ICC cohort compared to those in patients without PNI, whereas no difference in pan-neural markers such as ubiquitin carboxyl-terminal hydrolase isozyme L1 (UCHL1/PGP9.5) [25] (*p* = 0.292), beta-tubulin III (TUBB3) [26] (*p* = 0.656), synapsin (SYN) [27] (*p* = 0.612), and PNS biomarker vesicular acetylcholine transporter (VACHT) [28] (*p* = 0.266) was detected (Fig. 2b, c). Meanwhile, IHC staining consistently revealed that PNI exhibited positive staining for TH in TMA cohort (Fig. 2d), indicating that PNI originated from the SNS. As previously reported, neural transmitters such as, norepinephrine (NE) and acetylcholine (ACH) released from automatic nerves are transmitted and combined with corresponding receptors such as adrenergic receptors (ADRs), muscarinic acetylcholine receptors (CHRMs), and nicotinic acetylcholine receptors (CHRNs) in tumor cells or normal cells [23] (Fig. 2a). To detect the receptors involved in PNI, we analyzed the mRNA expression levels of ADRs in the ZS-ICC cohort. Surprisingly, significant upregulation of ADRB1 (*p* = 0.002) and ADRB3 (*p* = 0.029) expression and downregulation of ADRA2C (*p* = 0.006) expression were detected in patients with PNI, while no difference was found in other ADRs. (Fig. 2e). Furthermore, K–M analysis showed that ADRA2C (HR = 0.37, *p* < 0.001) played a protective role in the OS of patients with ICC after surgery, while ADRB1 (HR = 1.81, *p* = 0005) showed an adverse effect (Fig. S2a–c). In addition, as the main hydrolase of NE, MAO-A [29, 30] secreted from tumor cells was highly enriched in patients without PNI in TMA Cohort (*p* = 0.005) (Figs. 2f, g; S2d). Patients with high MAO-A level demonstrated improved OS and RFS (HR = 0.61, HR = 0.72, respectively) compared to those with low level of MAO-A (Fig. 2h, i).

### ICC patients with PNI were characterized by immune suppressive metastasis niche.

**Fig. 3:**
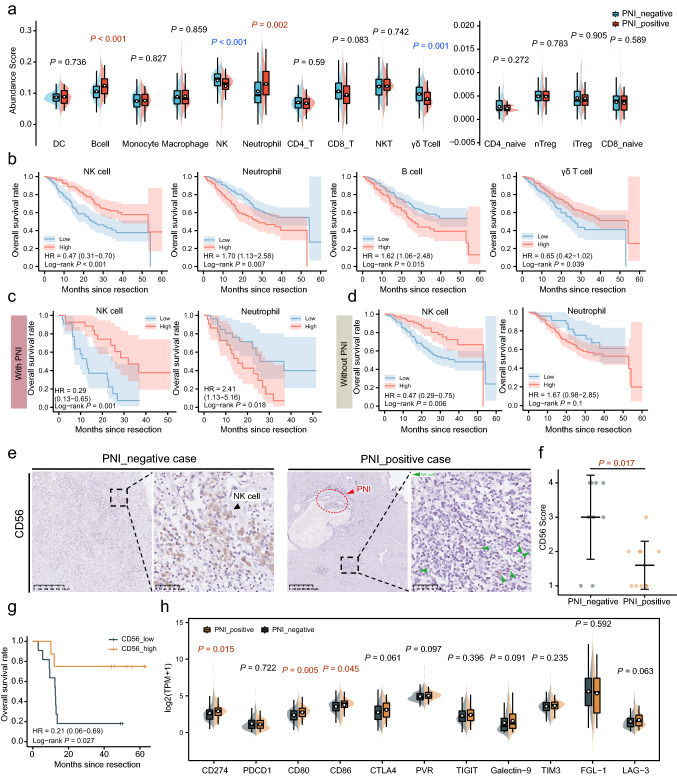
ICC patients with PNI were characterized by immune suppressive metastasis niche. (**a**) ssGSEA analysis of infiltrating immune cells of ZS-ICC cohort and unpaired t-test was used. (**b**) K–M analysis of OS among patients with different immune cell abundance score in ZS-ICC cohort. (**c**) K–M analysis of OS among PNI-positive patients with different infiltration of NK cells and neutrophils in ZS-ICC cohort. (**d**) K–M analysis of OS among PNI-negative patients with different infiltration of NK cells and neutrophils in ZSICC cohort. (**e**) Representative image of the CD56 IHC staining among ICC patients from cohort 4. (**f**) Comparison of CD56 expression score between PNI positive and PNI negative patients of cohort 4 (P = 0.017).Wilcoxon-test was used. (**g**) K–M analysis of OS between patients with high infiltration and low infiltration of CD56 positive NK cells in cohort 4. (CD56_low: IHC Score 1–2; CD56_high: IHC Score 3–4; HR = 0.21; P = 0.027). (**h**) The mRNA expression of immune checkpoints and relative ligands between patients with and without PNI in ZS-ICC cohort. Unpaired t-test was used

RNA-seq data from the ZS-ICC cohort showed a distinct gene expression pattern in patients with PNI (Fig. S3a, b). GO and KEGG analyses showed that upregulated genes in patients with PNI were majorly enriched in immune-associated pathways, such as neutrophil-mediated immunity, neutrophil degranulation, and leukocyte migration, while genes highly expressed in patients without PNI mostly focused on the regulation of neuron project development (Fig. S3c). To further elucidate the immune microenvironment in PNI-positive patients, ssGSEA was performed. Significantly decreased infiltration of NK cells (*p* < 0.001) and γδT cells (*p* = 0.001) and increased infiltration of B cells (*p* < 0.001) and neutrophils (*p* = 0.002) were found in patients with PNI in ZS-ICC cohort (Fig. 3a). Among these immune cells, the levels of NK cells and γδ T cells were positively correlated with the OS of ICC patients, whereas the levels of neutrophils and B cells were adversely correlated with OS (Fig. 3b). We further investigated the influence of these immune cells on the prognosis of patients with and without PNI. The results showed that patients with high level of NK cells had longer OS in either the PNI-positive group (HR = 0.29, *p* = 0.001) or the PNI-negative group (HR = 0.47, *p* = 0.006) (Fig. 3c). However, neutrophil levels only influenced the OS of patients with PNI (HR = 1.67, *p* = 0.1) (Fig. 3d). No statistical differences in B cells and γδT cells were found between patients with PNI (HR = 1.28, *p* = 0.535; HR = 0.53, *p* = 0.094) and those without PNI (HR = 1.43, *p* = 0.134; HR = 1.10, *p* = 0.693) (Fig. S3d, e). Similarly, decreased infiltration of NK cells (*p* = 0.017) was found in patients with PNI in cohort 4 (*n* = 19) (Fig. 3e, f), and patients with less infiltration of NK cells showed a worse prognosis (HR = 0.21; *p* = 0.027) (Fig. 3g). In addition, we also analyzed the expression of immune checkpoint molecules in the ZS-ICC cohort and found that the mRNA expression of PD-L1/CD274 (*p* = 0.015), CD80 (*p* = 0.005), and CD86 (*p* = 0.045) was distinctly elevated in patients with PNI (Fig. 3h). These data showed that ICC patients with PNI were accompanied with immune suppressive metastasis prone niche.

### ICC patients with PNI exhibited higher frequency of KRAS mutations.

**Fig. 4:**
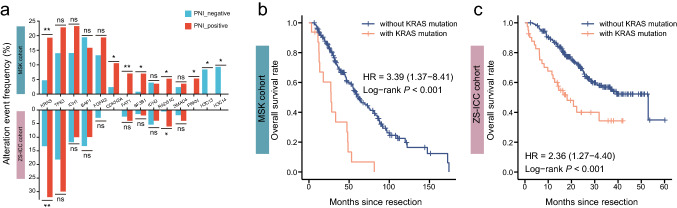
ICC patients with PNI exhibited higher frequency of KRAS mutations. (**a**) Bar plot of different mutation frequency genes in MSK and ZS-ICC cohorts (ns: P > 0.05; *: P < 0.05; **: P < 0.01). Chi-square test was used. (**b**) K–M analysis of OS between patients with and without KRAS mutations in MSK cohort (HR = 3.39, P < 0.001). (**c**) K–M analysis of OS between patients with and without KRAS mutations in ZS-ICC cohort (HR = 2.36, P < 0.001)

By comparing the genomic data of the MSK and ZS-ICC cohorts, a high alteration frequency of KRAS (*p* = 0.003; *p* = 0.003 respectively) and RAD51C (*p* = 0.028, *p* = 0.02 respectively) was observed in patients with PNI in both cohorts (Fig. 4a). K–M analysis revealed that patients with KRAS mutations also exhibited poor prognosis (HR = 3.39, *p* < 0.001; HR = 2.36, *p* < 0.001, respectively) (Fig. 4b, c), consistent with our previous report [31].

### ICC patients with PNI well responded to adjuvant therapy

**Fig. 5:**
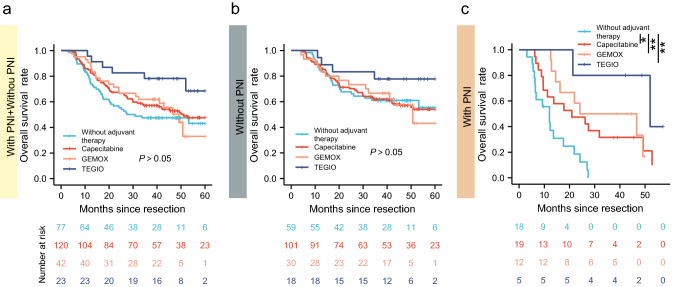
ICC patients with PNI well responded to adjuvant therapy. (**a**) K–M analysis of OS between patients with and without different adjuvant therapies in TMA cohort (log-rank P. adj ＞ 0.05). (**b**) K–M analysis of OS between PNI-negative patients with different adjuvant therapies and without adjuvant therapy in TMA cohort (log-rank P. adj ＞ 0.05). (**c**) K–M analysis of OS between PNI-positive patients with different adjuvant therapies and without adjuvant therapy in the TMA cohort (TEGIO vs. without adjuvant therapy: P. adj = 0.008; GEMOX vs. without adjuvant therapy: P. adj = 0.005; capecitabine vs. without adjuvant therapy: P. adj = 0.037; ns: P > 0.05; *: P < 0.05; **: P < 0.01). (**d**) K–M analysis of RFS between patients with and without different adjuvant therapies in TMA cohort (log-rank P. adj ＞ 0.05). (**e**) K–M analysis of RFS between PNI-negative patients with different adjuvant therapies and without adjuvant therapy in TMA cohort (log-rank P. adj ＞ 0.05). (**f**) K–M analysis of RFS between PNI-positive patients with different adjuvant therapies and without adjuvant therapy in the TMA cohrt (log-rank P. adj＞0.05)

We divided the TMA cohort into six subgroups according to different postoperative therapeutic strategies (Table 2). K–M analysis was used to compare the OS of patients with or without adjuvant therapy. No statistical difference in OS was observed between the patients who received adjuvant therapy and those who did not (Fig. 5a). We further investigated the role of adjuvant therapy in patients with PNI and found that these patients gained significant OS benefits from adjuvant chemotherapy, such as capecitabine (*p* = 0.037), GEMOX (*p* = 0.005), and TEGIO (*p* = 0.008), while patients without PNI did not (*p* > 0.05) (Fig. 5b, c).

**Table 2:** Summary of adjuvant therapies of TMA cohort

	PNI		Total
	Negative	Positive	
GEMOX	30	12	42
Capecitabine	101	19	120
TEGIO	18	5	23
Multiple^a^	13	5	18
TACE	24	5	29
Without adjuvant therapy	59	18	77
Total	245	64	309

*GEMOX* gemcitabine and oxaliplatin, *TEGIO* tegafur, gimeracil, oteracil and porassium capsules, *TACE* transcatheter arterial chemoembolization

^a^Treatment regimens changed in the follow-up records

## Discussion

Our study revealed that PNI could be considered an unfavorable prognostic factor for patients with ICC post-surgery, and an indicator of well response to adjuvant therapy. Furthermore, our results showed that PNI in patients with ICC was mainly from SNS. Moreover, our study is the first to decipher the immune suppressive metastasis prone niche of ICC patients with PNI, which was characterized by decreased infiltration of NK cells, increased infiltration of neutrophils and elevated expression of immune check points' ligands. In addition, PNI positive ICC patients are accompanied with higher frequency of KRAS mutation. These data indicate that ICC with PNI exhibits special microenvironment, potentially causing the invasion and metastasis of ICC.

PNI is considered as a mechanistic feature associated to tumor metastasis and a marker of poor prognosis in several cancers [7–12]. In the present study, our results provide sufficient evidence to support the notion that PNI is a reliable marker for predicting the prognosis of patients with ICC, based on two independent cohorts. Recent studies have demonstrated that neuromodulation plays an important role in several pathological processes, including tumor metastasis and remodeling of the immune microenvironment [3, 6, 14, 32]. Nerve domination largely depends on the context of the type of tumor. For example, PNI reprograms the immune microenvironment through cholinergic signaling in PDAC [6], while PNI in head and neck cancer is associated with the adrenergic nerve [33, 34]. In the present study, PNI in patients with ICC exhibited positive staining for TH, indicating that it originated from the SNS. ADRs have been widely reported to be involved in cancers recently [35]. As the main neural transmitter released from SNS, NE can bind with ADRs on the surface of tumor cells, immune cells, and other stromal cells, and exert biological action [23]. In our study, the expression of ADRA2C, ADRB1, and ADRB3 in patients with PNI evidently differed from that in patients without PNI, and patients with increased ADRA2C and decreased ADRB1 showed better prognosis. ADRB1 expressed on tumor cells has been identified as a biomarker for breast cancer [36]. ADRA2C, mainly expressed on the pre-synapse neurons, is thought to act as an inhibitory modulator of the sympathetic nervous system [37]. The overexpression of ADRB1 and downregulation of ADRA2C probably hyperactivate SNS signaling in ICC patients with PNI, resulting in PNI-mediated poor prognosis. Additionally, as the main hydrolase of NE [29], MAO-A was enriched in PNI-negative patients, indicating that it inhibited SNS in PNI. A recent study reported that MAO-A suppressed HCC metastasis by inhibiting adrenergic signaling [38]. Interestingly, MAO-A promotes prostate cancer cell PNI through SEMA3C/PlexinA2/NRP1-cMET signaling [39]. This contradictory conclusion largely resulted from the fact that SNS was not the main constituent of PNI in prostate cancer [40]. Altogether, NE from SNS might activate multiple ADRs in ICC patients with PNI, and cancer cell-derived MAO-A might inactivate NE from SNS, synergistically involved in PNI-mediated tumor progression.

The association between SNS and the immune system has been documented over the last several decades [41]. Recent studies have shown complicated but important interactions between immune cells and neurons in tumor tissues, called ‘neuro-immune unit’ [15]. In this study, patients with PNI showed an immune suppressive metastasis prone niche with increased infiltration of B cells and neutrophils and decreased infiltration of NK cells and γδ T cells. The protective role of NK cells was confirmed in both PNI-negative and PNI-positive groups. In mammary adenocarcinoma, acute stress could activate SNS, resulting in the suppression of NK cell activity and tumor metastasis [42]. SNS also induces the suppression of NK cell cytotoxicity in rats [43]. Thus, we hypothesized that the involvement of SNS in ICC with PNI inhibits the infiltration of NK cells, partly contributing to the poor prognosis of patients. In cancers, tumor-associated neutrophils (TANs) have emerged as an important component of the tumor microenvironment, which can not only be a part of tumor-promoting inflammation by driving angiogenesis, extracellular matrix remodeling, metastasis, and immunosuppression, but can also mediate antitumor responses by directly killing tumor cells and participating in cellular networks that mediate antitumor resistance [44]. Zhou. et al. reported that TANs and macrophage interactions contribute to ICC progression by activating STAT3 [45]. Consistently, we observed an adverse relationship between neutrophils and the prognosis of ICC patients with PNI. Interestingly, we did not detect any influence of neutrophils on the survival of patients without PNI. As reported previously, local SNS signaling promotes neutrophil infiltration during acute inflammation [46]. A recent study also demonstrated that stress hormones such as NE and adrenaline cause rapid release of proinflammatory S100A8/A9 proteins by neutrophils, leading to early relapse in lung cancer and ovarian cancer post operation [47]. In this study, we observed an increased infiltration of neutrophils in ICC patients with PNI. These indicate an interaction between PNI and neutrophils. Elevated expression of PD-L1, CD80, and CD86 was observed in patients with PNI. Our previous study showed that PD-L1 was a predictive marker for immunotherapy in ICC [48], implying that ICC patients with PNI might benefit more from anti-PD1/PD-L1 therapy. Meanwhile, the interaction of CTLA-4 with CD80 or CD86 could inhibit human T-cell activation [49], indicating that the inhibition of CD80 and CD86 in PNI-positive patients might activate T cells and restore antitumor immune reactions. Surprisingly, targeting the CD80/CD86 costimulatory pathway also directs microglia towards a repair phenotype and promotes axonal outgrowth [50]. In conclusion, neurons can release neural transmitters and other cytokines to bind the receptors on immune cells and tumor cells, regulating their functions, while immune cells and tumor cells could release several neurotrophic factors to promote the axons extension. The interaction among neurons, immune cells and malignancies synthetically cause the occurrence of PNI and the immune suppressive metastasis prone niche. Thus, further research is needed to define whether PNI or immune regulation is the cause or result in such a complex microenvironment.

Consistent with a retrospective study of 86 ICC patients [51], we found that the alteration frequency of KRAS in the PNI-positive group was higher than that in the PNI-negative group, partly contributing to the poor prognosis of patients with PNI. Li. et al. also reported that KRAS mutations are associated with PNI in colon cancer [52]. KRAS, a Kirsten ras oncogene homolog from the mammalian ras gene family, encodes a protein that is a member of the small GTPase superfamily [53]. Ras proteins bind GDP/GTP and possess intrinsic GTPase activity, playing an important role in GPCR (G-protein combined receptors) mediated pathways like adrenergic signaling [53]. We hypothesized that the mutation of KRAS hyperactive the downstream pathways of ADRs, contributing to the development of PNI. Additionally, syndecan-2 (SDC-2) promotes perineural invasion and cooperates with K-ras to induce an invasive pancreatic cancer cell phenotype [54]. What’s more, the M2 splice isoform of PK (PKM2) was found to regulate neural invasion of hilar cholangiocarcinoma (HC) via regulation of SDC2 [55]. These indicate SDC-2 may be an intermediator between KRAS mutation and PNI in ICC. This may provide valuable information for the curation of treatment strategies of ICC patients with PNI. However, additional evidence is needed to illustrate direct interactions between KRAS mutations and PNI.

Clinical trials have demonstrated that adjuvant chemotherapy, including TEGIO and capecitabine, is beneficial for patients with biliary tract cancers, including ICC [56]. In this study, we retrospectively observed that patients with PNI gained OS benefits from adjuvant chemotherapy, while no improvement in OS was found in PNI-negative patients. The same phenomenon has been reported in colon [57], rectal [58], prostate [59], and oral cancers [60]. This result suggests that PNI could be a credible predictor for adjuvant therapy in ICC. Regrettably, the mechanism underlying the relationship between PNI and adjuvant therapy remains unclear.

## Conclusion

Our study deciphered that patients with PNI showed a poor prognosis after surgery but a good response to adjuvant chemotherapy. In addition, we illustrated that ICC patients with PNI were accompanied with higher frequency of KRAS mutation and an immune suppressive metastasis prone niche characterized by decreased NK cell, increased neutrophil and elevated expression of immune check points’ ligands. However, further investigations are needed to explore the molecular mechanisms of PNI in ICC.

## Data sharing statement

Data are available from the authors upon reasonable request.

## Supplementary Information

Below is the link to the electronic supplementary material.

Supplementary file1 (PDF 825 KB)

Supplementary file2 (PDF 19072 KB)

Supplementary file3 (PDF 3638 KB)

Supplementary file4 (PDF 196 KB)

Supplementary file5 (PDF 171 KB)

Supplementary file6 (PDF 135 KB)

## Data Availability

Data and materials are available on request.

## Abbreviations

PNI: Perineural invasion
ICC: Intrahepatic cholangiocarcinoma
GO: Gene ontology
KEGG: Kyoto Encyclopedia of Genes and Genomes
ssGSEA: Single sample Gene Set Enrichment Analysis
TMA: Tissue microarray
HCC: Hepatocellular carcinoma
PDAC: Pancreatic ductal adenocarcinoma
OS: Overall survival
RFS: Relapse-free survival
TNM: Tumor-node-metastases
AJCC: American Joint Committee on Cancer
IHC: Immunohistochemistry
H&E: Hematoxylin–eosin
MAO-A: Monoamine oxidase A
TH: Tyrosine hydroxylase
OD: Optical density
SNS: Sympathetic nerve system
PNS: Parasympathetic nerve system
UCHL1: Ubiquitin carboxyl-terminal hydrolase isozyme L1
TUBB3: Tubulin beta-3 chain
SYN: Synapsin
VACHT: Vesicular acetylcholine transporter
NE: Norepinephrine
ACH: Acetylcholine
ADRs: Adrenergic receptors
CHRMs: Muscarinic acetylcholine receptors
CHRNs: Nicotinic acetylcholine receptors
TME: Tumor microenvironment
NK: Natural killer
TEGIO: Tegafur, Gimeracil and Oteracil potassium capsules
GEMOX: Gemcitabine + Oxaliplatin
ICBs: Immune check point blockers
PD1: Programmed death 1
PD-L1: Programmed death ligand-1
CTLA-4: Cytotoxic T-lymphocyte associated protein 4
TIGIT: T cell immunoreceptor with Ig and ITIM domains
PVR: Poliovirus receptor
TIM3: T cell immunoglobulin domain and mucin domain-3
FGL-1: Fibrinogen-like protein-1
LAG-3: Lymphocyte-activation gene 3
DC: Dendrite cell
Treg: Regulatory T cell
TPM: Transcripts Per Kilobase of exon model per Million mapped reads
