# Supplementary material for: The clinical implications and molecular features of intrahepatic cholangiocarcinoma with perineural invasion
Source: Hepatol Int. 2022 Nov 22;17(1):63–76. doi: 10.1007/s12072-022-10445-1 (PMC9895046; doi:10.1007/s12072-022-10445-1)
Supplement: Supplementary file 1 — Supplementary file1 (PDF 825 KB) [file 12072_2022_10445_MOESM1_ESM.pdf]

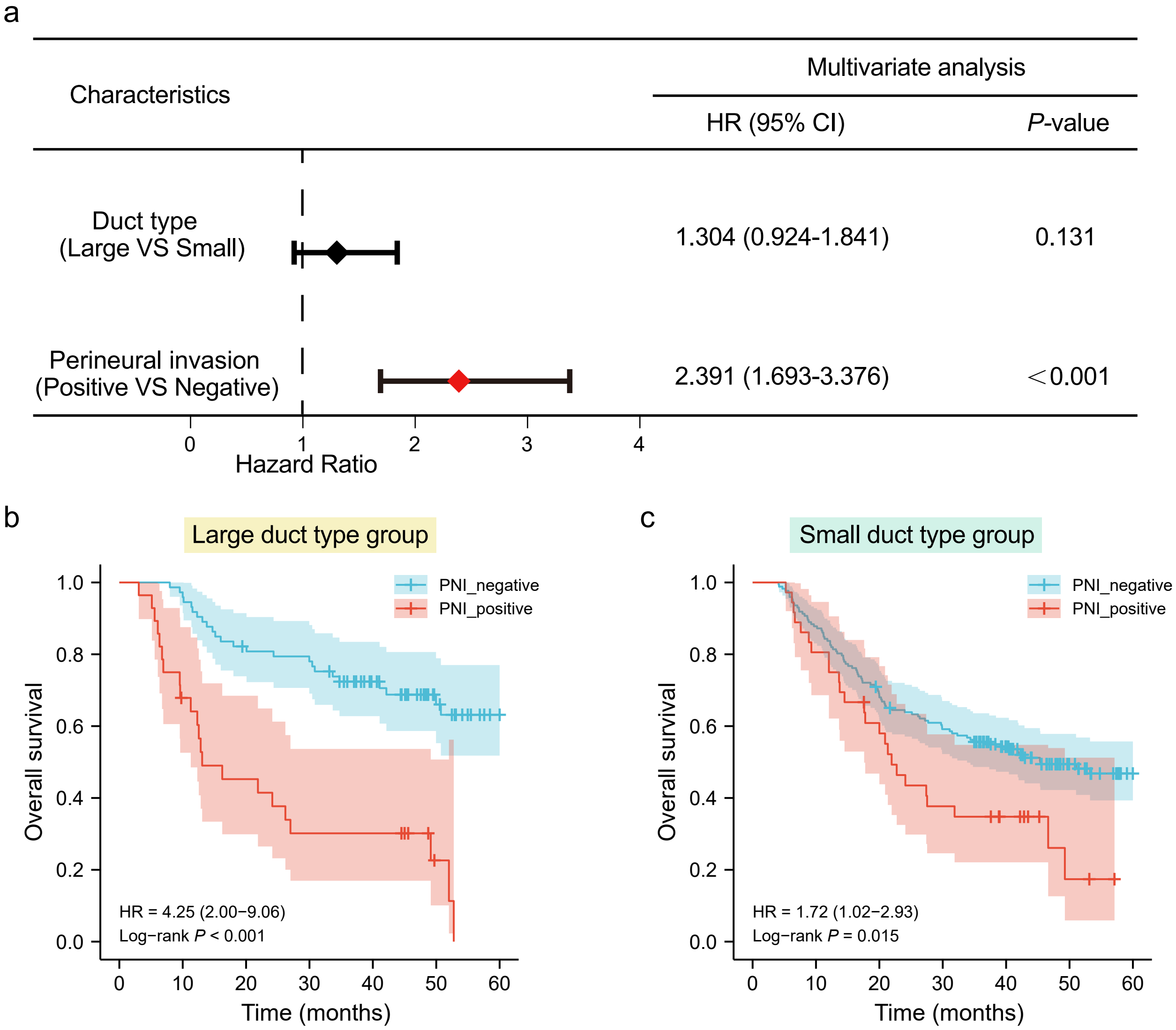

Supplementary figure1:

a. Multivariate analysis of duct type and PNI in TMA cohort.
b. K-M analysis of OS between patients with PNI and without PNI in the large duct type group of TMA cohort (HR=4.25, *P*< 0.001).
c. K-M analysis of OS between patients with PNI and without PNI in the small duct type group of TMA cohort (HR=1.72, *P*=0.015).
