# Supplementary material for: The clinical implications and molecular features of intrahepatic cholangiocarcinoma with perineural invasion
Source: Hepatol Int. 2022 Nov 22;17(1):63–76. doi: 10.1007/s12072-022-10445-1 (PMC9895046; doi:10.1007/s12072-022-10445-1)
Supplement: Supplementary file 2 — Supplementary file2 (PDF 19072 KB) [file 12072_2022_10445_MOESM2_ESM.pdf]

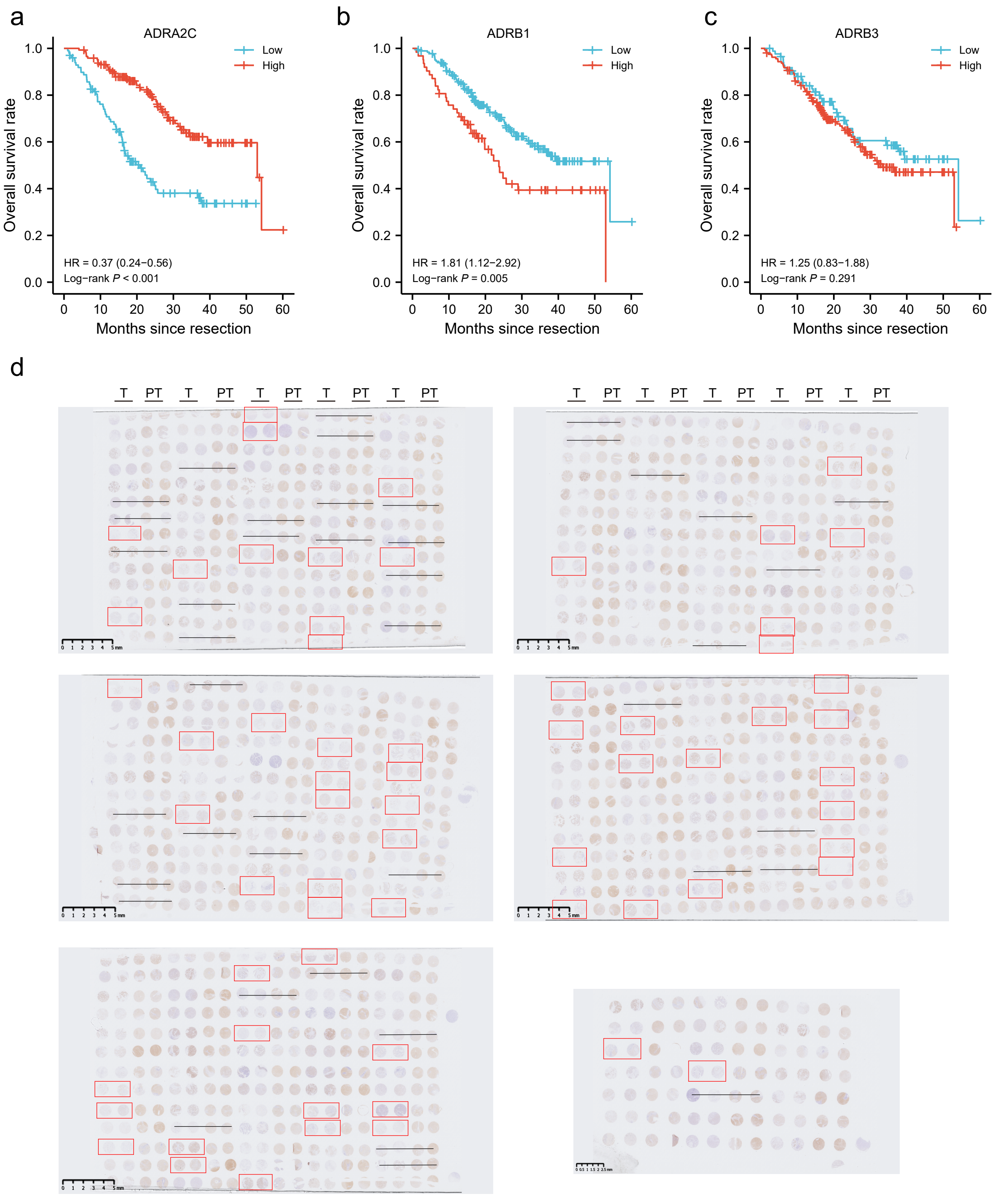

Supplementary figure 2:

a. K-M analysis of OS between ICC patients with different expression of ADRA2C in ZS-ICC cohort (HR=0.37, P < 0.001).
b. K-M analysis of OS between ICC patients with different expression of ADRB1 in ZS-ICC cohort (HR=1.81, P =0.005).
c. K-M analysis of OS between ICC patients with different expression of ADRB3 in ZS-ICC cohort (HR=1.25, P =0.291).
d. Global exhibition of IHC of MAOA in TMA cohort (from the left to the right: 4 points for each patient, 2 of tumor tissue and 2 of para-tumor tissue; —— : patients did not meet the recurit criteria and was excluded; ▭ : tumor tissue of PNI positive cases; T: tumor; PT: para-tumor).
