# Supplementary material for: The clinical implications and molecular features of intrahepatic cholangiocarcinoma with perineural invasion
Source: Hepatol Int. 2022 Nov 22;17(1):63–76. doi: 10.1007/s12072-022-10445-1 (PMC9895046; doi:10.1007/s12072-022-10445-1)
Supplement: Supplementary file 3 — Supplementary file3 (PDF 3638 KB) [file 12072_2022_10445_MOESM3_ESM.pdf]

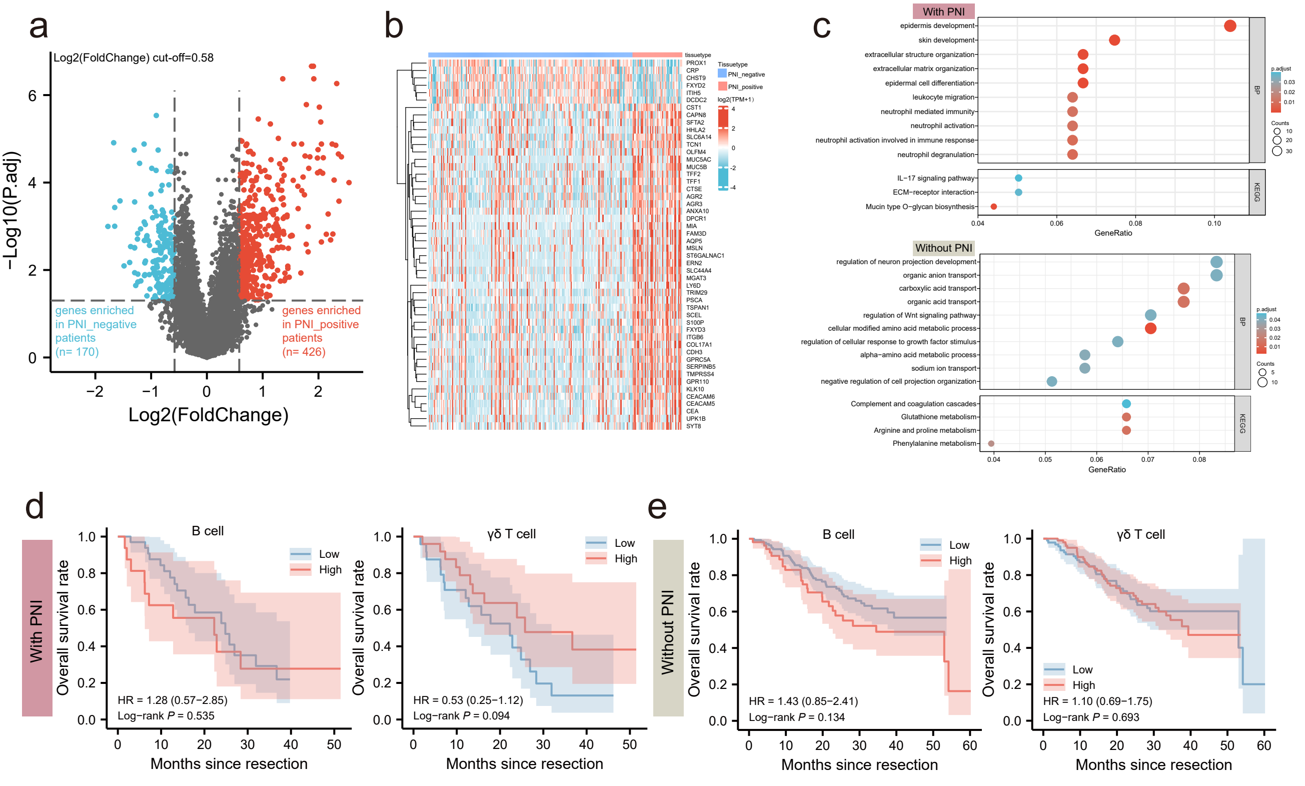

Supplementary figure 3:

a. Volcano plot of differentially expressed genes between patients with and without PNI in the ZS-ICC cohort (P.adj cut-off=0.05; |log2Foldchange| cut-off=0.58; 426 genes enriched in the PNI_positive group; 170 genes enriched in the PNI_negative group).
b. Heatmap of the top 50 differentially expressed genes between patients with and without PNI in the ZS-ICC cohort (clustered by genes).
c. GO and KEGG analyses of genes enriched in patients with PNI and withou PNI in ZS-ICC cohort.
d. K–M analysis of OS among PNI-positive patients with different infiltration of B cells and γδ T cell in ZS-ICC cohort.
e. K–M analysis of OS among PNI-negative patients with different infiltration of B cells and γδ T cell in ZS-ICC cohort.
