# Supplementary material for: The clinical implications and molecular features of intrahepatic cholangiocarcinoma with perineural invasion
Source: Hepatol Int. 2022 Nov 22;17(1):63–76. doi: 10.1007/s12072-022-10445-1 (PMC9895046; doi:10.1007/s12072-022-10445-1)
Supplement: Supplementary file 4 — Supplementary file4 (PDF 196 KB) [file 12072_2022_10445_MOESM4_ESM.pdf]

Table S1: Baseline demographics and clinicopathological variables among patients of ICC with and without PNI in MSK cohort.

		PNI		*P* value
		negative	positive	
Age (year)	≤65	67	29	0.894
	＞65	62	28	
Sex	Female	73	33	0.868
	Male	56	24	
Hepatolithiasis	Negative	NA	NA	NA
	Positive	NA	NA	
HBV infection	Negative	121	56	0.279#
	Positive	8	1	
ALT (U/L)	≤75	NA	NA	
	＞75	NA	NA	
AFP (ng/mL)	≤20	NA	NA	
	＞20	NA	NA	
CA19-9 (U/mL)	≤37	43	10	0.018*
	＞37	41	26	
	NA	45	21	
Tumor size (cm)	≤5	56	23	0.697
	＞5	73	34	
Tumor number	Single	NA	NA	NA
	Multiple	NA	NA	
Duct type	Small	118	39	＜0.001*
	Large	6	15	
	Indeterminate	3	3	
	NA	2	0	
Lymph node invasion	Negative	109	40	0.024*
	Positive	20	17	
Microvascular invasion	Negative	NA	NA	
	Positive	NA	NA	
TNM stage	I/II	NA	NA	
	III	NA	NA	
Tumor differentiation	Low	39	16	0.766
	Moderate/High	90	41	
Adjuvant therapy	No	90	24	＜0.001*
	Yes	38	31	
	NA	1	2	

#： Fisher's exact test; *: $P < 0.05$; Abbreviations: HBV: hepatitis B virus; ALT: alanine aminotransferase; AFP: alpha fetoprotein; CA19-9: carbohydrate antigen199.
