# Supplementary material for: The clinical implications and molecular features of intrahepatic cholangiocarcinoma with perineural invasion
Source: Hepatol Int. 2022 Nov 22;17(1):63–76. doi: 10.1007/s12072-022-10445-1 (PMC9895046; doi:10.1007/s12072-022-10445-1)
Supplement: Supplementary file 5 — Supplementary file5 (PDF 171 KB) [file 12072_2022_10445_MOESM5_ESM.pdf]

Table S2: Baseline demographics and clinicopathological variables among patients of ICC with and without PNI in ZS-ICC cohort.

		PNI		*P* value
		negative	positive	
Age (year)	≤65	130	33	0.938
	>65	73	19	
Sex	Female	93	18	0.146
	Male	110	34	
Hepatolithiasis	Negative	194	49	0.714[#]
	Positive	9	3	
HBV infection	Negative	140	47	0.002
	Positive	63	5	
ALT (U/L)	≤75	192	43	0.008*
	>75	11	9	
AFP (ng/mL)	≤20	179	50	0.090
	>20	24	2	
CA19-9 (U/mL)	≤37	101	11	0.000*
	>37	102	41	
Tumor size (cm)	≤5	90	21	0.608
	>5	113	31	
Tumor number	Single	NA	NA	NA
	Multiple	NA	NA	
Duct type	Small	NA	NA	NA
	Large	NA	NA	
Lymph node invasion	Negative	164	35	0.036*
	Positive	39	17	
Microvascular invasion	Negative	122	22	0.021*
	Positive	81	30	
TNM stage	I/II	138	21	0.000*
	III/IV	65	31	
Tumor differentiation	Low	NA	NA	NA
	Moderate/High	NA	NA	
Adjuvant therapy	No	135	38	0.365
	Yes	68	14	

#: Fisher's exact test; *: $P < 0.05$; Abbreviations: HBV: hepatitis B virus; ALT: alanine aminotransferase; AFP: alpha fetoprotein; CA19-9: carbohydrate antigen199;
