# Supplementary material for: The clinical implications and molecular features of intrahepatic cholangiocarcinoma with perineural invasion
Source: Hepatol Int. 2022 Nov 22;17(1):63–76. doi: 10.1007/s12072-022-10445-1 (PMC9895046; doi:10.1007/s12072-022-10445-1)
Supplement: Supplementary file 6 — Supplementary file6 (PDF 135 KB) [file 12072_2022_10445_MOESM6_ESM.pdf]

Supplementary Table 3: Comparison of the basic information of MSK and TMA cohort.

		cohort		*P* value
		MSK	TMA	
Age (year)	⩽65	96	221	<0.001*
	>65	90	88	
Sex	Female	106	118	<0.001*
	Male	80	191	
Hepatolithiasis	Negative	NA	254	NA
	Positive	NA	55	
HBV infection	Negative	177	58	<0.001*
	Positive	9	251	
ALT (U/L)	⩽75	NA	287	NA
	>75	NA	22	
AFP (ng/mL)	⩽20	NA	290	NA
	>20	NA	19	
CA19-9 (U/mL)	⩽37	53	164	0.097
	>37	67	145	
	NA	66	0	
Tumor size (cm)	⩽5	79	171	0.005*
	>5	107	138	
Tumor number	Single	NA	236	NA
	Multiple	NA	73	
Duct type	Small	157	208	<0.001*
	Large	21	101	
	Indeterminate	6	0	
	NA	2	0	
Lymph node invasion	Negative	149	263	0.152
	Positive	37	46	
Microvascular invasion	Negative	NA	242	NA
	Positive	NA	67	
TNM stage	I/II	NA	231	NA
	III/IV	NA	78	
Tumor differentiation	Low	55	43	<0.001*
	Moderate/High	131	266	
Perineural invasion	Negative	129	245	0.013*
	Positive	57	64	
Adjuvant therapy	No	114	77	<0.001*
	Yes	69	232	
	NA	3	0	

*: $P < 0.05$; Abbreviations: HBV: hepatitis B virus; ALT: alanine aminotransferase; AFP: alpha fetoprotein; CA19-9: carbohydrate antigen199;
